# The banana code—natural blend processing in the olfactory circuitry of *Drosophila melanogaster*

**DOI:** 10.3389/fphys.2014.00059

**Published:** 2014-02-20

**Authors:** Marco Schubert, Bill S. Hansson, Silke Sachse

**Affiliations:** Department of Evolutionary Neuroethology, Max Planck Institute for Chemical EcologyJena, Germany

**Keywords:** *Drosophila*, gas chromatography, *in vivo* calcium imaging, olfaction, blend coding, insect antennal lobe

## Abstract

Odor information is predominantly perceived as complex odor blends. For *Drosophila melanogaster* one of the most attractive blends is emitted by an over-ripe banana. To analyze how the fly's olfactory system processes natural blends we combined the experimental advantages of gas chromatography and functional imaging (GC-I). In this way, natural banana compounds were presented successively to the fly antenna in close to natural occurring concentrations. This technique allowed us to identify the active odor components, use these compounds as stimuli and measure odor-induced Ca^2+^ signals in input and output neurons of the *Drosophila* antennal lobe (AL), the first olfactory neuropil. We demonstrate that mixture interactions of a natural blend are very rare and occur only at the AL output level resulting in a surprisingly linear blend representation. However, the information regarding single components is strongly modulated by the olfactory circuitry within the AL leading to a higher similarity between the representation of individual components and the banana blend. This observed modulation might tune the olfactory system in a way to distinctively categorize odor components and improve the detection of suitable food sources. Functional GC-I thus enables analysis of virtually any unknown natural odorant blend and its components in their relative occurring concentrations and allows characterization of neuronal responses of complete neural assemblies. This technique can be seen as a valuable complementary method to classical GC/electrophysiology techniques, and will be a highly useful tool in future investigations of insect-insect and insect-plant chemical interactions.

## Introduction

The natural environment displays a myriad of vital cues coded in complex odor blends, which often are composed of a large number of single odor components. Information processing of simultaneous input regarding several different odor compounds forming a specific and behaviorally relevant representation is so far poorly understood. Hereby, a question of general importance arises: Does the olfactory system process and encode simultaneously occurring components as blend-specific information? And does this representation evolve over the different levels of olfactory processing? We addressed these questions by analyzing physiological responses to a natural odor blend and its single odor components in the antennal lobe (AL) of the vinegar fly *Drosophila melanogaster*.

**Figure 1:**
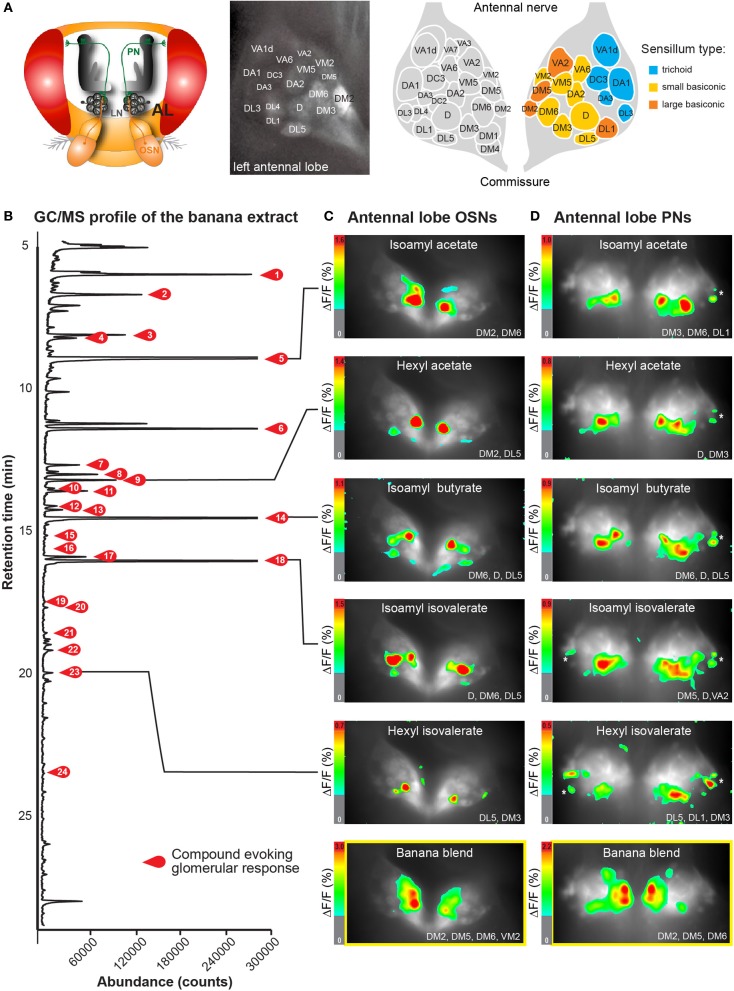
**Neuronal activity patterns of banana compounds in the fly antennal lobe using combined gas chromatography and functional imaging (GC-I). (A)** *Left*, schematic illustrating the *Drosophila* olfactory system. Odor information detected by the antenna is conveyed by olfactory sensory neurons (OSN: red) to the antennal lobe (AL). OSNs converge in specific glomeruli and synapse onto local interneurons (LNs: gray) and projection neurons (PNs: green). *Middle*, baseline fluorescence of G-CaMP in axon termini of OSNs in the AL with anatomical identification of individual glomeruli. The antennal nerve enters the imaged region at the top; the AL commissure is at the bottom. *Right*, schematic AL map viewed from the angle used for imaging experiments. Colored glomeruli (*n* = 17) could reliably be identified; colors correspond to their sensillum input. **(B)** GC/MS profile of a banana extract revealing single compounds that compose the banana headspace extract (see Table 1 for peak identity of active compounds corresponding to the red number tags). **(C,D)** Pseudocolor rendering of Ca^2+^ responses of OSNs **(C)** and PNs **(D)** to different banana extract compounds and the complete banana blend. Images represent Δ*F*/*F*_0_ (%) superimposed onto raw fluorescence images according to the scale bars on the left. Black lines serve to link the corresponding peaks **(B)** to the evoked activity patterns. White asterisk shows cell body activity in the lateral cell clusters. The most active glomeruli are indicated in the lower right corner.

*Drosophila* detects odor molecules with two olfactory organs, the maxillary palps and the antennae. Different types of olfactory sensilla house olfactory sensory neurons (OSNs) carrying different types of odorant receptors (ORs) (Hallem and Carlson, 2006; Vosshall and Stocker, 2007; Hansson et al., 2010). OSNs can either be narrowly tuned or respond to a broad range of structurally similar odor ligands (De Bruyne et al., 2001; Hallem and Carlson, 2006; Pelz et al., 2006; Stensmyr et al., 2012). From the antenna the information is conveyed to the ALs, the first relay station of the olfactory pathway (Figure 1A). Each group of OSNs, carrying the same type of OR, converge onto one or a few specific olfactory glomeruli (Gao et al., 2000; Vosshall et al., 2000; Couto et al., 2005; Fishilevich and Vosshall, 2005; Silbering et al., 2011). Each AL comprises about 50 glomeruli, which represent structural and functional units that shape and modulate the odor information on its way to higher processing centers (Laissue et al., 1999; Couto et al., 2005; Galizia and Sachse, 2010). Within the glomeruli, OSNs exchange information with local interneurons (LNs) and projection neurons (PNs) by excitatory and inhibitory synaptic crosstalk (Wilson, 2011). Since each OSN type targets its own specific glomerulus, the detection of odor molecules leads to a specific mosaic of glomerular activity patterns (Fiala et al., 2002; Ng et al., 2002; Wang et al., 2003; Silbering et al., 2008).

When two odors are processed simultaneously by the olfactory system, odor mixture interactions might occur. These can result in either suppression or synergism leading to a reduced or an enhanced mixture response compared to single component responses (Akers and Getz, 1993; Duchamp-Viret et al., 2003; Deisig et al., 2006; Silbering and Galizia, 2007; Rospars et al., 2008; Kuebler et al., 2011; Münch et al., 2013). Most recent physiological studies on odor mixture processing have mainly focused on binary or quaternary mixtures with monomolecular synthesized odor compounds because of application advantages (Deisig et al., 2006; Silbering and Galizia, 2007; Grossman et al., 2008; Fernandez et al., 2009; Deisig et al., 2010). In order to analyze neuronal processing of a complex naturally occurring mixture and subsequently to identify its individual odor components, we combined the experimental advantages of gas chromatography with functional imaging, subsequently called functional GC-I, as previously established for the mouse olfactory system (Lin et al., 2006). Here, we examine one of the most attractive food sources and breeding places for *Drosophila melanogaster*, an over-ripe banana (Sturtevant, 1921; Lachaise and Silvain, 2004), in its natural composition. Using functional GC-I enabled us to capture and identify potentially relevant odor components of a banana headspace extract. In addition this technique allowed us to use these components as stimuli in close to natural relative proportions, and monitor odor-induced Ca^2+^ dynamics *in vivo*. To do so, we expressed the genetically encoded Ca^2+^ indicator G-CaMP at different processing levels of the *Drosophila* AL. We show that the banana extract consists of 24 active compounds that induce clear neuronal activity in AL input and output neurons. We demonstrate that mixture interactions within a natural banana blend are very rare and occur only at the AL output level resulting in a surprisingly linear blend representation. We further show, however, that individual glomerular responses are significantly modified by the neural circuitry in the AL from the input to the output level resulting in a modulated odor representation. This modulation leads to a higher similarity between the representations of individual components in relation to the complete banana blend. Such processing mechanism might tune the olfactory system in a way to categorize key components with their naturally occurring odor source to enhance the detection of suitable food sources.

## Materials and methods

### Animals

We used 6–10 days old female vinegar flies (*Drosophila melanogaster*) raised on conventional cornmeal-agar-molasses medium under L:D/12:12, *RH*=70% and 25°C. Transgenic lines used: Orco-GAL4 (Larsson et al., 2004), GH146-GAL4 (Stocker et al., 1997), UAS-G-CaMP1.6 (Nakai et al., 2001; Wang et al., 2003). Flies were dissected for optical imaging as described (Stökl et al., 2010; Strutz et al., 2012). Briefly, flies were anesthetized on ice, fixed with the neck onto a Plexiglas stage using a copper plate (Athene Grids). The head was glued at the stage with colophony resin (Royal Oak, Rosinio) and the antennae were gently pulled forward with a fine metal wire (H.P. REID co. inc., USA). Polyethylene foil was attached on the head and sealed to the cuticle with two-component silicone (KwikSil, WPI). A small hole was cut through the foil and cuticle. Immediately after opening of the head, the brain was bathed with Ringer solution (130 mM NaCl, 5 mM KCl, 2 mM MgCl_2_, 2 mM CaCl_2_, 36 mM sucrose, 5 mM Hepes, [pH 7.3]). Removal of trachea and glands allowed optical access to the ALs.

### Optical imaging

We used a Till Photonics imaging system with an upright Olympus microscope (BX51WI) equipped with a 20 × Olympus objective (XLUM Plan FL 20x/0.95W). A Polychrome V provided light excitation (475 nm) and a filter set ensured passage of only relevant wavelengths (excitation: SP500, dicroic: DCLP490, emission: LP515). The emitted light was captured by a CCD camera (Sensicam QE, PCO AG) with a symmetrical binning of 4 (1.25 × 1.25 μm/pixel). For each measurement a series of 300 frames was taken (1 Hz, GC-I run time 3.5–8.5 min). A low sample rate of 1 Hz prevented the fluorescent Ca^2+^ sensor from bleaching over the 300 images taken. To assure that we did not lose any information by the low imaging sample rate of 1 Hz we also tested a 2 Hz frequency showing that no additional response peaks were registered (data not shown).

### Data analysis

All imaging data were analyzed using custom software written in IDL (ITT Visual Information Solutions). For anatomical identification of glomeruli we compared the glomerular organization of the ALs with an available standard atlas (Laissue et al., 1999) as we have previously described in detail (Stökl et al., 2010). For data analyses, the activity of individual glomeruli was taken as an area of 5 × 5 pixels per glomerulus. A bleaching correction was applied for each frame (300 frames per imaging sequence) by subtracting the median fluorescence from each pixel. An automated movement correction compensated for movement artifacts between frames during the imaging sequence. To achieve a comparable standard for the calculation of the relative fluorescence changes (Δ*F*/*F*_0_), we defined the background fluorescence (*F*_0_) as the mean of 10 successive frames before the stimulation with the extract components. This background was then subtracted for each glomerulus during the whole sequence of 300 frames, so that basal fluorescence has been normalized to zero. The calcium responses of each identified glomerulus were synchronized to the GC banana profile data by using isoamyl acetate and butyl butyrate peaks as prominent orientation points. A data matrix was generated for the fluorescence changes of each identified glomerulus over each of the 300 frames imaged. Glomerular responses were normalized within each animal to the strongest glomerulus response measured which was set to 100%. After identification of all 70 odor components of the banana extract, we included in our analysis only those fluorescent changes that corresponded to the component peaks in the GC run for each glomerulus. We then determined those components that induced a calcium signal resulting in 24 active components (Table 1) and used these for further analysis. Hence our data matrix represents population vectors that are defined by the identity of a glomerulus in one dimension and the calcium signal for each of the 24 odor components in the other dimension.

**Table 1:** **Identified and physiological active banana compounds**.

**Order of compounds**	**Retention time (min)**	**Kovats indices**	**Absolute peak height (MS/GC counts)**	**CAS**	**Compound**
1	6.172	770	1985038	110-19-0	Isobutyl acetate
2	6.878	802	425162	105-54-4	Ethyl butyrate
3	8.273	850	486014	626-38-0	2-Pentyl acetate
4	8.398	854	93711	108-64-5	Ethyl isovalerate
5	9.127	878	7707050	123-92-2	Isoamyl acetate
6	11.537	956	3759896	539-90-2	Isobutyl butyrate
7	12.799	996	2450746	109-21-7	Butyl butyrate
8	13.127	1007	1944566	589-59-3	Isobutyl isovalerate
9	13.352	1014	4112522	142-92-7	Hexyl acetate
10	13.619	1023	2718249	72237-36-6	4-Hexenyl acetate
11	13.721	1026	3100127	60415-61-4	2-Pentyl butyrate
12	14.255	1044	2318335	5921-82-4	2-Heptyl acetate
13	14.381	1048	1640472	109-19-3	1-Butyl isovalerate
14	14.812	1062	13284855	106-27-4	Isoamyl butyrate
15	15.281	1077	373711	89155-38-4	2-Pentylvalerianate
16	18.827	1095	63619	n/a	MIX: n-pentyl butyrate/n-butyl valerate
17	16.024	1101	2283078	27625-35-0	Isoamyl 2-Methyl butyrate
18	16.261	1110	11475565	659-70-1	Isoamyl isovalerate
19	17.507	1152	181278	105-79-3	Isobutyl hexanoate
20	17.565	1154	339344	2050-09-1	Isoamyl valerate
21	18.686	1193	1282073	2639-63-6	Hexyl butyrate
22	19.292	1214	942710	39026-94-3	2-Heptyl butyrate
23	20.081	1243	2062633	10032-13-0	Hexyl isovalerate
24	23.677	1378	32112	n/a	Isomer of octenyl butyrate

Listed are the banana compounds following the retention times needed to be detected by the FID. Compounds were identified by Kovats retention indices (non-isotherm) for temperature programmed methods.

Normalized responses within identified glomeruli were compared using Student's *t*-tests (unpaired two-tailed distribution). In order to analyze the proximity of our odor representations to the 24 active components and the banana blend in a putative neural space, we regarded each odor representation as a vector in a multidimensional space, in which each dimension is represented by a glomerulus. We used the relative fluorescence changes (Δ*F*/*F*_0_) in single frames (i.e., corresponding to each one of the 24 components) for each identified glomerulus and calculated the Euclidean distances between each single odor component and the banana blend to quantify the pattern proximity. Furthermore, we applied principal coordinates analysis including all glomeruli that we could identify at both processing levels (*n* = 10) in order to visualize the pattern similarity in a lower-dimensionality space formed of a subset of highest-variance components (Deisig et al., 2010). Statistical analyses were performed with the software GraphPad Instat and PAST.

### Odor extract

Banana extracts were produced from commercially available ripe bananas. Cut bananas including the skin were placed into an oven-bag (Toppits© Roasting-bags, www.toppits.de) which was perforated with air holes on one side and connected to a Super-Q filter (50 mg, Analytical Research Systems, Inc.) on the other side. A pump (Casella Apex lite) sucked banana odor laden air for 4 h with a constant flow rate (1 l/min.) over the filter, which was eluted with hexane (300 μl) afterwards and the extract was stored at −20°C until use. Silicon tubing and Teflon© connectors were used to avoid contamination. In control experiments extracts coming from bananas of different age (degree of fermentation) showed similar GC profiles in terms of individual components and only partially differences with respect to component concentration (data not shown). Extracts from older bananas typically provided higher concentrations of molecules emerging in the first half of the GC profile. Despite major concentration differences, the glomerular activity was qualitatively almost concentration independent for the used extracts.

### Gas chromatography

We injected 2 μl of banana extract into an Agilent 6890N GC (Agilent Technologies). Separated extract components were leaving the GC via a heated and flexible transfer line (GC outlet). The transfer line head was mounted with a Pasteur pipette in which the components were injected. A constant purified and humidified airstream carried the stimuli through the pipette to the fly antenna. For GC parameter control and data acquisition an external computer running the commercial software GC ChemStation (Agilent Technologies) was used. GC banana blend data collected during imaging (5 min, sample rate of 1200 Hz) were synchronized with the imaging data for detailed comparisons. Subsequent GC/MS (5975B inert XL MSD, Agilent Technologies) analysis was used for identification of all active components.

The Agilent 6890N GC (Agilent Technologies) was running the injector in splitless mode (250°C) using helium as a makeup/carrier gas which did not induce any glomerular responses. At the end of a HP-5 low/non-polar column (flow rate: 2 ml/min; column length: 30 m, inner diameter: 0.32 mm, inside coating: 0.25 μm, thick film of 5% phenyl methyl siloxane and 95% methyl siloxane) the sample stream was split in two parts (1:1), one leading to a flame ionization detector (FID, detector temp.: 310°C) and the other leaving the GC via a heated and flexible transfer line (GC outlet). During each run the GC oven and transfer line temperature was synchronized, ramped from 40°C (1 min) at 20°C/min to 300°C. The transfer line head at the end (300°C constantly) was mounted with a Pasteur pipette (length: 12cm) in which the separated stimuli components were injected via the GC outlet.

### Odor puff stimulation

After each functional GC-I run the animals were exposed to odor puff stimulations with the banana extract, the solvent hexane and an air control, while glomerular AL responses were optically recorded. A stimulus controller (CS-55, Syntech) provided a continuous air flow (0.5 l/min) in which odor injection could be applied via two disposable Pasteur pipettes. For odor stimulation the air stream switched from a blind Pasteur pipette to the stimulus pipette in which the filter paper was odor laden for 2 s. The banana extract was applied in the same concentration as the GC fractionated banana components to allow for subsequent comparison.

## Results

### Neuronal representation of banana odor components

By combining the experimental advantages of gas chromatography and Ca^2+^ imaging, we measured the representation of single banana compounds and the complete banana blend at different levels of olfactory processing in the *Drosophila* olfactory system. The volatile collection of an over ripe banana was injected into a GC, where it was separated into more than 70 individual components (Figure 1B). In this way, banana odor components could be presented successively to the fly antenna in naturally occurring concentrations. An imaging capture rate of 1 Hz allowed us to measure the responses to each single component that emerged from the GC, since components were separated by at least 1 s. Identification of components that induced a significant increase in the intracellular calcium concentration ([Ca^2+^]_*i*_) in the AL was subsequently performed using GC-mass spectrometry (Table 1) and was well in line with compounds earlier identified in banana headspace extracts (Shiota, 1993; Jordán, 2001; Stensmyr et al., 2003).

Primarily, we measured the representation of single banana odor compounds and the complete banana blend in input neurons, i.e. the axonal terminals of OSNs in the AL (Figure 1C, Supplementary Movie). Using the binary GAL4-UAS transcriptional system (Brand and Perrimon, 1993), we genetically expressed the Ca^2+^-sensitive reporter G-CaMP (Nakai et al., 2001) in the majority of OSNs employing Orco-GAL4 (Wang et al., 2003). Since the AL morphology with its glomerular structure is invariant and clearly visible, we could identify individual glomeruli in every animal using the available 3D atlas of the *Drosophila* AL (Laissue et al., 1999). This identification enabled us to assign odor-evoked Ca^2+^ responses to 17 glomeruli (52% of all glomeruli labeled by Orco-GAL4) and hence to correlate those to specific sensilla and OR types on the antenna (Figures 1A,C) (Hansson et al., 2010). We observed significant odor-evoked Ca^2+^ responses to 24 out of the 70 banana extract compounds. Activation of OSNs by single banana components and by the complete blend resulted in specific combinatorial patterns of activated glomeruli.

Secondly, we examined the representation of the single banana compounds and the complete blend at the next processing level, the dendrites of AL output neurons. To achieve this, we expressed G-CaMP in the majority of PNs using the enhancer trap line GH146-GAL4 (Figure 1D) (Stocker et al., 1997). Similar to the OSN recordings, the odor-evoked responses could be reliably assigned to 15 identified glomeruli (41% of all glomeruli labeled by GH146-GAL4). Since GH146-GAL4 does not label glomeruli VM5 and VA6, these could not be characterized at the PN level. We observed specific odor-evoked Ca^2+^ responses for the same 24 banana compounds as detected during the OSN recordings. The complete banana blend induced a broad but, nevertheless, specific pattern of activated glomeruli at both processing levels (Figures 1C,D lowest panel).

### Comparison between input and output representation

**Figure 2:**
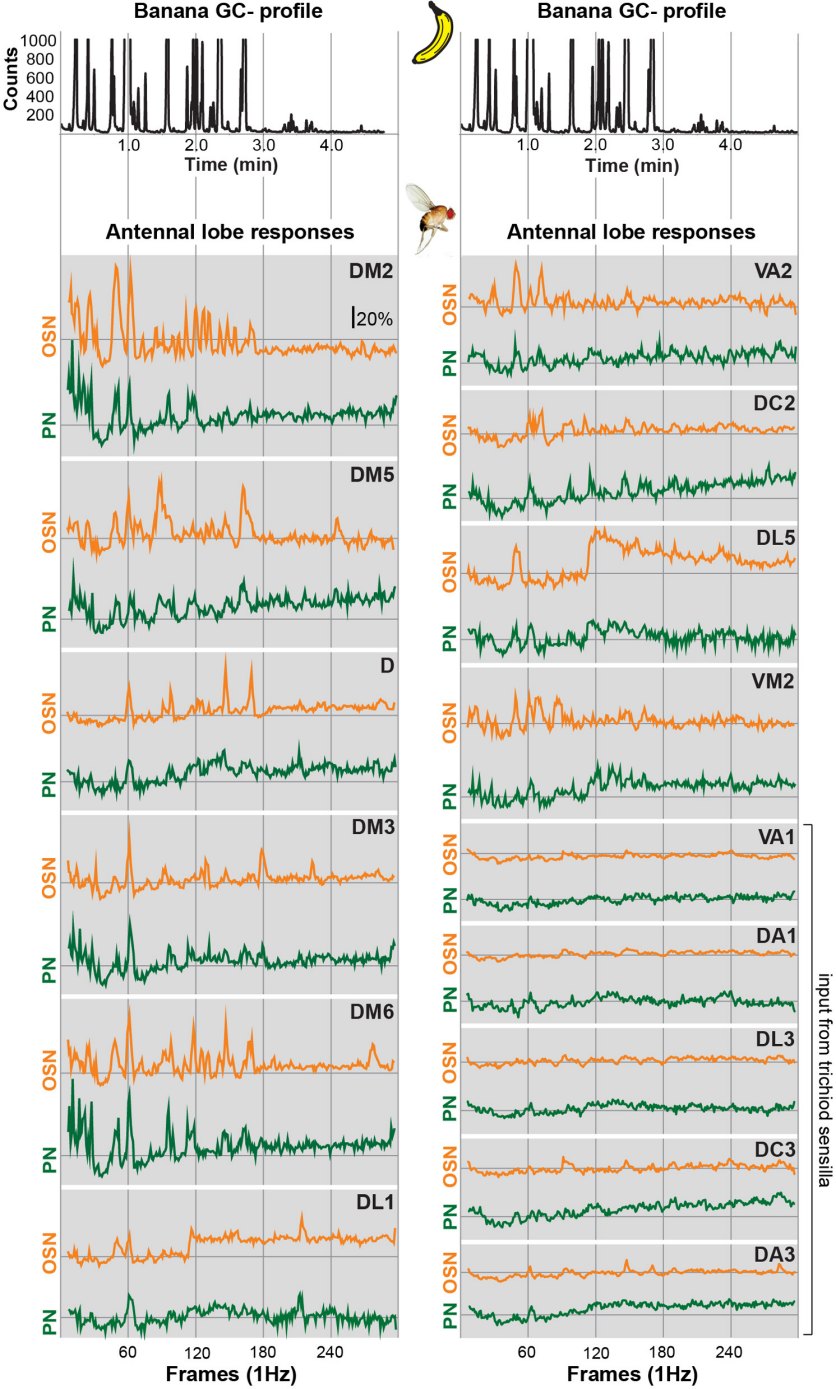
**Glomerular response traces of input and output neurons to banana extract stimulation.** *Top*, GC profile (black line) of the banana extract. *Below*, synchronized time courses of Ca^2+^ dynamics are shown for the OSN (orange traces) and PN level (green traces) averaged across five flies, respectively. Traces are given for all individual glomeruli that could be identified at both processing levels. The sample rate (frames/second) was 1 Hz. The whole measurement lasted for 5 min. Time traces represent the percentage of intensity changes compared to background activity (Δ*F*/*F*).

**Figure 3:**
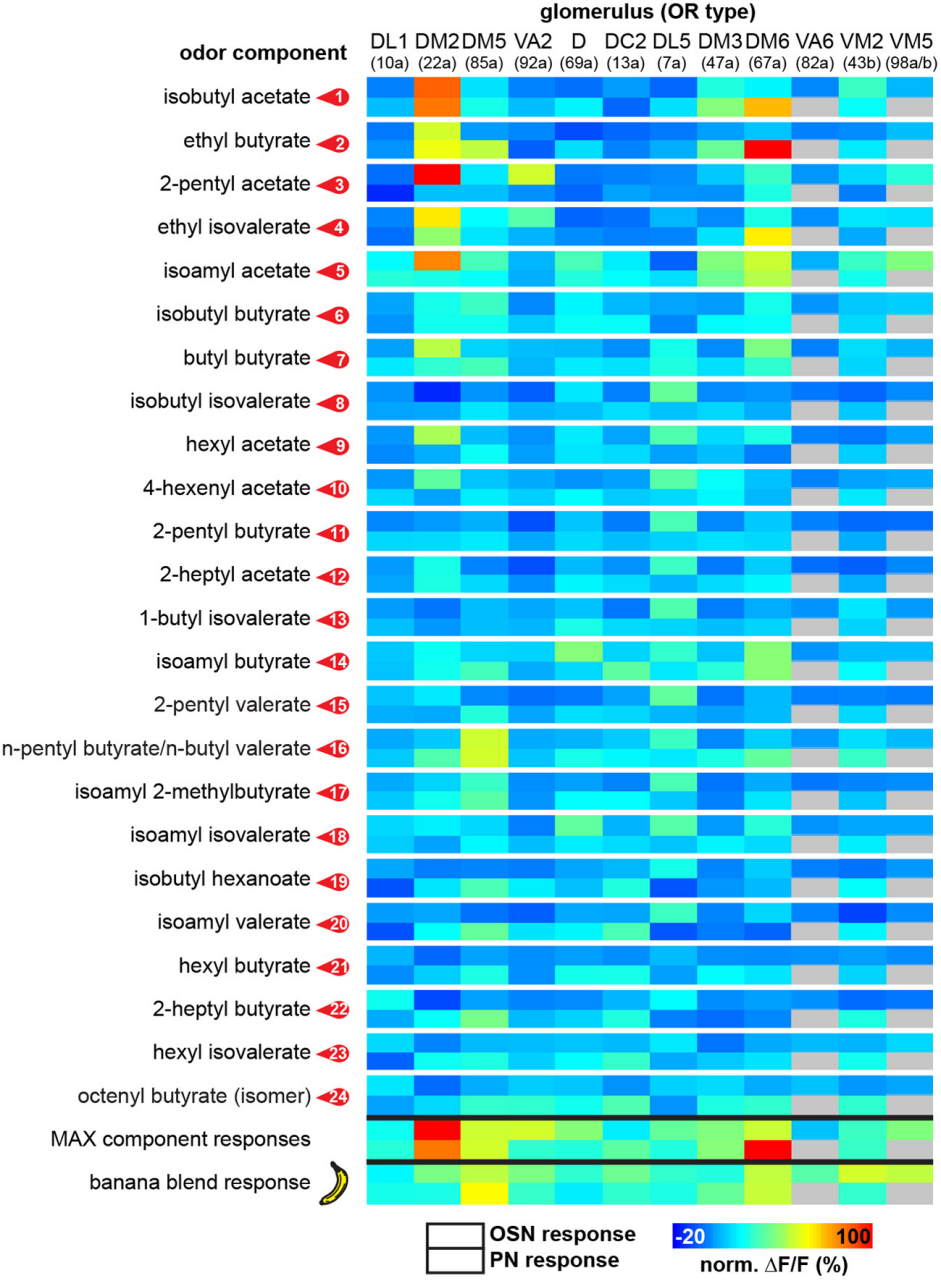
**Functional map of odor-evoked glomerular activation to banana compounds.** The odor responses of 12 glomeruli are shown for each identified banana compound at the OSN (upper box) and the PN level (lower box) as a heat map. Each data point is the median glomerular response from five flies. The last two rows represent glomerular responses to the measured complete banana blend and the strongest responses calculated for each component as a prediction for the banana blend response. For each individual glomerulus the corresponding odorant receptor input is given in brackets.

In order to allow a comparison between the two processing levels, we synchronized the response profiles of OSNs and PNs and aligned them to the chromatograms (Figure 2) using characteristic component landmarks such as isoamyl acetate and isoamyl butyrate (#5 and #14 in Figure 1B). Notably, glomerulus-specific time traces of OSNs and PNs showed similar, but not identical odor response properties indicating that the odor-evoked responses are modulated from the input to the output level. Several glomeruli revealed a broad response profile while others, in particular glomeruli receiving input from trichoid sensilla, showed only sparse or no activity at all. To simplify the recorded Ca^2+^ dynamics, we quantified the odor-evoked response intensities for all identified glomeruli and summarized these as a heat map for each individual banana compound and the blend response (Figure 3). We excluded here glomeruli receiving input from trichoid sensilla, since they did not show any significant responses to the tested banana components. The most prominent AL responses were recorded in glomeruli DM2 and DM6. Interestingly, odors with a similar retention time and therefore similar chemical properties activated a similar combination of glomeruli confirming a previous imaging study in honeybees (Sachse et al., 1999). The banana blend itself evoked a very broad response pattern (Figure 3, last row). Comparison between OSN and PN response intensities shows again that the odor representations are different between the two processing levels.

**Figure 4:**
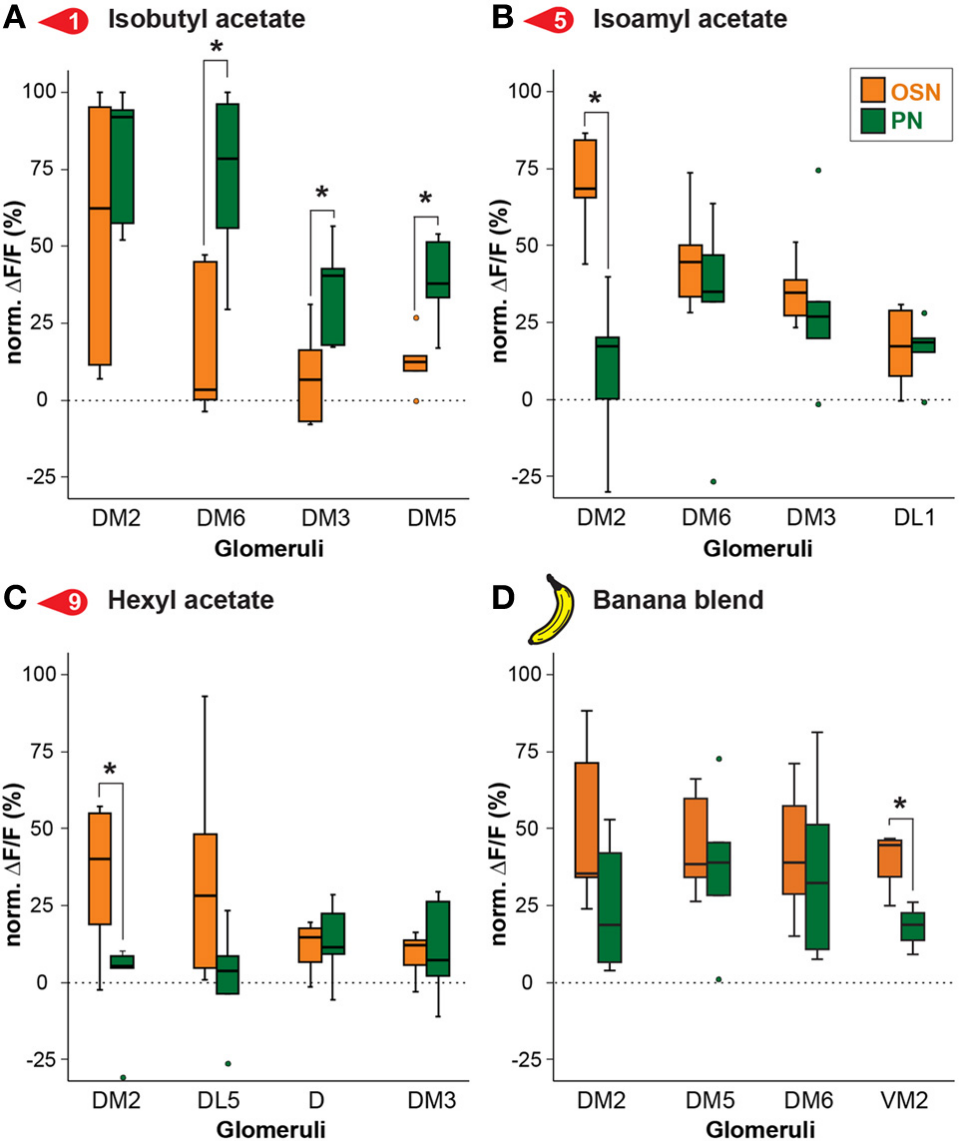
**Odor response modulation between OSN and PN level.** Examples of glomerular responses to three identified banana compounds **(A–C)** and the banana blend **(D)** are shown as a comparison between the OSN (orange) and PN (green) level for the four most active glomeruli per component. Box plots here and in Figure 5 represent the median value (horizontal line inside the box), the interquartile range (height of the box, 50% of the data are within this range) and the minimum and maximum value (whiskers) of each experimental group. Circles depict outliers with values that were more than 1.5 times the interquartile range from the lower or upper quartile. Fluorescence values represent the average percentage of intensity changes compared to background activity (Δ*F*/*F*_0_, *n* = 5). Responses were normalized to highest calcium response in each animal over all odors before averaging. Significant differences are indicated with asterisks (^*^p < 0.05; unpaired *t*-test).

To further examine this difference, we compared in detail the response of the strongest activated glomeruli between OSNs and PNs. Figure 4 depicts comparisons of odor responses between the two processing levels for three exemplary banana extract components and the complete banana blend. As already visible in the heat map (Figure 3), several glomeruli showed significantly higher responses at the PN level than at the OSN level as shown, e.g., for the odor isobutyl acetate (Figure 4A). However, we also observed that some odors induced a significantly reduced PN response in comparison to the OSN response as shown for glomerulus DM2 (Figures 4B,C). Interestingly, when comparing the input and output glomerular responses to the complete banana blend, we found a significant reduction in the PN response of glomerulus VM2 (Figure 4D). The observed signal modulation between input and output neurons shows a strong diversity of odor information transfer in a glomerulus-specific manner. However, we only observed a significant signal modulation in 8% of glomerular responses, while the majority of glomeruli showed almost identical signals between the two processing levels.

### Linearity of blend representation

**Figure 5:**
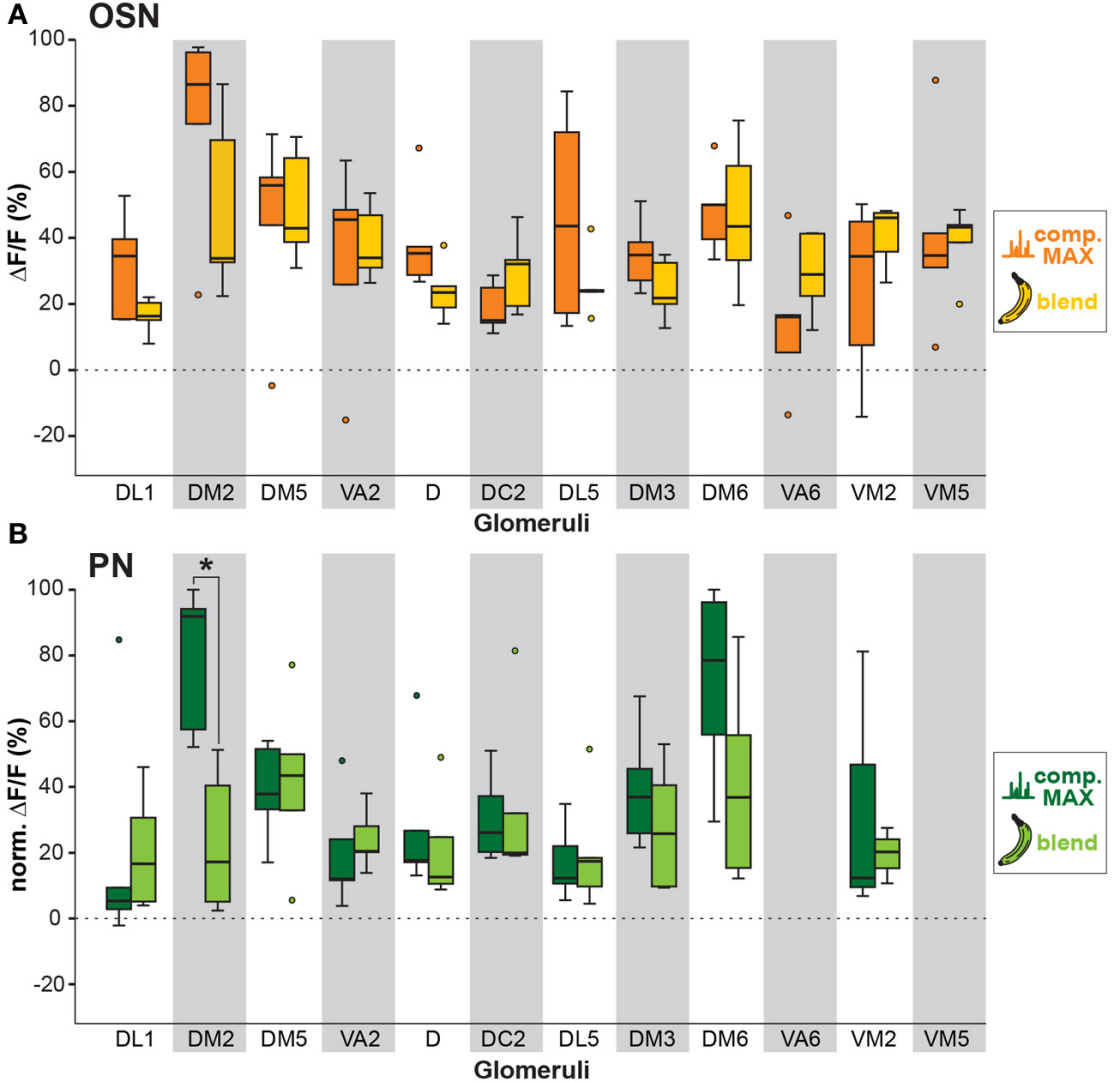
**Comparison between individual compound and banana blend representation. (A)** Orange boxplots represent the strongest OSN responses to any of the extract compounds, while yellow boxplots represent the response to the banana blend in the corresponding glomeruli. Differences between boxplots were not significantly different (unpaired *t*-test, *n* = 5). **(B)** Same analysis as in **(A)** at the PN level. Dark green boxplots represent the strongest PN component responses, whereas light green boxplots represent blend responses. Glomeruli VA6 and VM5v are not labeled by GH146-GAL4 and could therefore not be analyzed at the PN level. Glomerulus DM2 shows a significant lower response to the blend than to the strongest single component (^*^p < 0.05; unpaired *t*-test, *n* = 5).

We next analyzed if the banana odor blend was linearly represented in the AL as predicted from the glomerular activation patterns induced by single components, or if the AL network was modulating the blend response to something different than predicted, implying non-linear blend effects. Since the concentration of the single components in the GC run is approximately equal as during the puff stimulation, we expect that the individual glomerular responses to the blend should be as strong as the maximal glomerular response to the single odor components (MAX component response). Interestingly, when we used this rather conservative approach to calculate the blend response, we could very well predict the actual measured blend response (Figure 3, last two rows). This observation is further supported by a direct comparison between the maximal component responses and the blend response which reveals no significant differences for any of the glomeruli measured at their input site (Figure 5A). The same analysis at the PN level shows a similar picture: The responses of most glomeruli did not differ between blend and single component stimulation except for glomerulus DM2 whose activity was significantly reduced during the actual blend application (Figure 5B). Hence, blend interactions of the banana blend are rare and occur only at the AL output level leading to a surprisingly linear blend representation.

### Banana blend representation is modulated between processing levels

**Figure 6:**
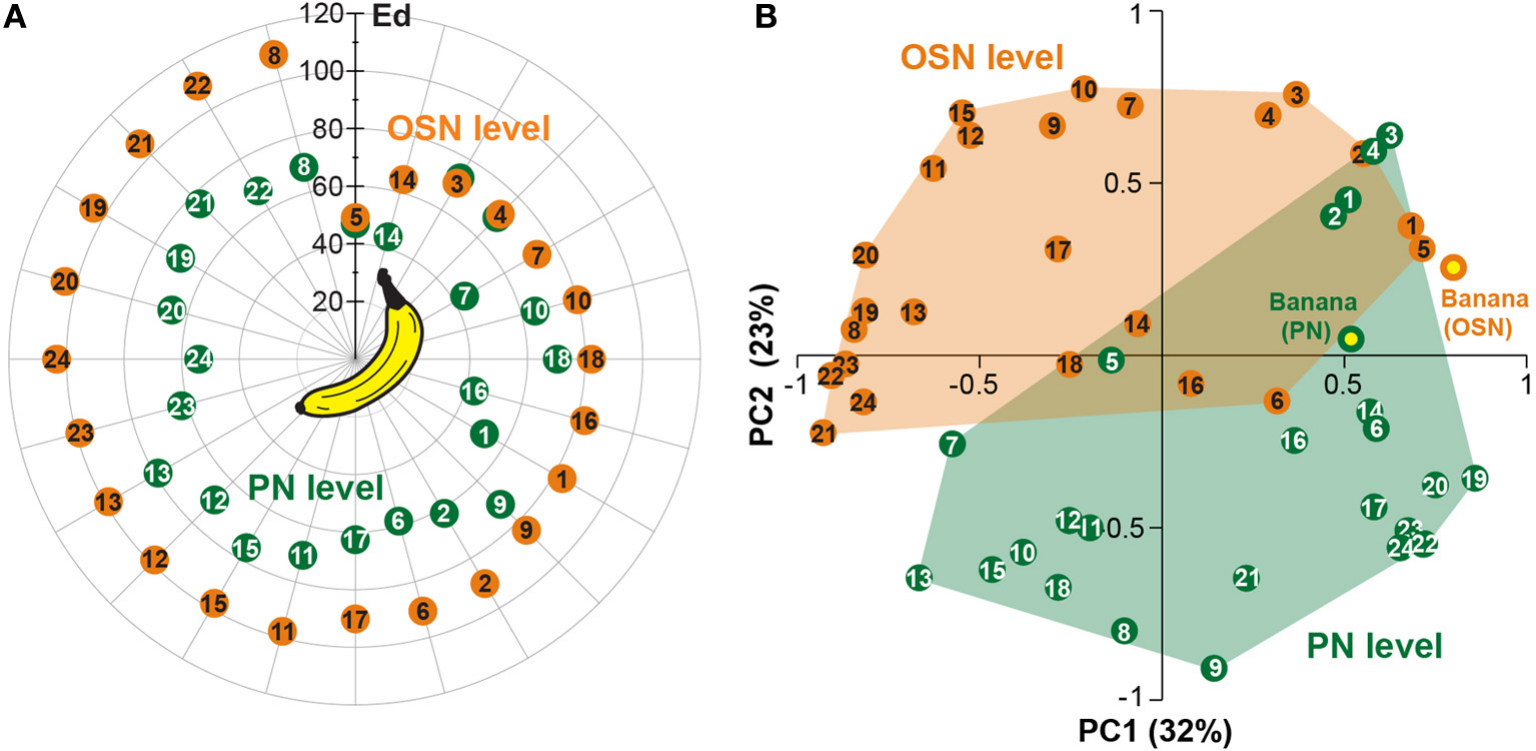
**Odor component and blend similarity. (A)** Similarity between single components and the banana blend responses at the OSN (orange number tags) and PN (green number tags) level. The Euclidean distances (*Ed*) between component and blend responses were calculated and represented as the distance between the number tags and the banana center in a polar plot. Short distances between the tags and the center represent high, longer distances reveal low similarities. The similarities between component and blend representations are lower for all, except three components, at the PN level than at the OSN level. **(B)** Principal coordinates analysis of the individual component and blend responses at the OSN (orange) and PN (green) level. The odor representations of the two processing levels form significantly distinct clusters (^***^p < 0.001, One-Way ANOSIM, Bray-Curtis). The representation of the banana blend is located among the single component representations at the PN level, while it is outside of the cluster at the OSN level.

In order to analyze if a single odor component could be as representative as an over-ripe banana to a vinegar fly, we determined which one of the single components represented best the banana blend. To judge the similarity we calculated the Euclidean distances between component and blend response patterns and identified components producing the most similar response patterns compared to the banana evoked pattern (Table 2). Both OSN and PN similarity rankings include similar key components as 2-pentyl acetate (#3), ethyl isovalerate (#4), isoamyl acetate (#5) and isoamyl butyrate (#14). Interestingly, when we compared the Euclidean distances between the blend representation and the single components we observed that these were significantly lower at the PN level than at the OSN level (on average 0.89 for OSNs versus 0.63 for PNs; ^***^p < 0.001, paired *T*-test, Table 2, Figure 6A). This modulation leads to a higher similarity between the representations of individual components in relation to the complete banana blend.

**Table 2:** **Component and blend similarity**.

**Order of compounds**	**OSN *Ed***	**PN *Ed***	**Compound**
5	52, 41	47, 48	Isoamyl acetate
14	65, 28	45, 67	Isoamyl butyrate
3	70, 87	72, 35	2-Pentyl acetate
4	71, 90	68, 88	Ethyl isovalerate
7	73, 23	45, 46	Butyl butyrate
10	79, 85	66, 44	4-Hexenyl acetate
18	81, 91	70, 98	Isoamyl isovalerate
16	82, 65	42, 16	Mix: n-pentyl butyrate/n-butyl valerate
1	83, 12	51, 85	Isobutyl acetate
9	85, 41	73, 04	Hexyl acetate
2	89, 16	61, 96	Ethyl butyrate
6	89, 30	58, 31	Isobutyl butyrate
17	90, 30	63, 05	Isoamyl 2-methyl butyrate
11	96, 98	69, 11	2-Pentyl butyrate
15	97, 18	74, 22	2-Pentylvalerianate
12	98, 29	67, 69	2-Heptyl acetate
13	98, 55	78, 99	1-Butyl isovalerate
23	98, 78	61, 80	Hexyl isovalerate
24	103, 67	53, 07	Isomer of octenyl butyrate
20	105, 42	66, 96	Isoamyl valerate
19	105, 90	71, 49	Isobutyl hexanoate
21	107, 48	74, 75	Hexyl butyrate
22	108, 55	67, 73	2-Heptyl butyrate
8	108, 86	68, 54	Isobutyl isovalerate

Lineup of the Euclidean distances (Ed) between component and blend responses.

Next, we applied principal coordinates analysis to visualize the odor evoked ensemble activity to all banana compounds in relation to the blend pattern at both processing levels and found three main results (Figure 6B): First, the odor-evoked responses of the two levels form significantly distinct clusters supporting the observed activity pattern modulation between OSNs and PNs (^***^p < 0.001, One-Way ANOSIM, Bray-Curtis). Second, the different component representations of OSNs and PNs are spread over a similar sized odor space. And third, the blend representation appears outside of the cluster at the OSN level, while it is located among the single component representations at the PN level. Hence, the representation of individual banana components is shifted toward the blend representation at the output level. This change in physiological representation is leading to a higher similarity between the banana components in relation to the banana blend.

## Discussion

With the functional GC-I technique we established an experimental tool that solves the dilemma of using either a natural odor source (without knowing its exact component composition) or a synthetic odor blend (without knowing the natural concentrations) as an experimental stimulus. A fundamental question appearing when comparing functional GC-I runs and conventional blend stimulation is the use of comparable concentrations in both situations. Since we used identical extract concentrations that induced comparable glomerular response intensities for both odor applications, we conclude that both stimulations provided comparable stimulus concentrations to the animal's antenna. Another critical point is to verify that the animal was not adapted during the GC-I measurement. The single banana components were separated by at least 1 s when they emerged from the GC column, resulting in an inter-stimulus-interval (ISI) of 1 Hz. Since it has been shown that even PNs can reliably follow an ISI of up to 2 Hz without being adapted (Brown et al., 2005), and since we applied very low odor concentrations, we assume that the flies were not adapted during the GC-I recording.

### Signal modulation during the transition from input to output neurons

Our finding that some PN component responses were significantly reduced, while others were increased compared to the response intensity of OSNs (Figures 3, 4) proposes an interplay of inhibitory and excitatory processes caused by the neuronal network within the AL. The neuronal substrate for glomerulus-specific modulation is provided by inhibitory and excitatory LNs that have been characterized and suggested to be involved in the processing mechanisms of the AL (Wilson and Laurent, 2005; Shang et al., 2007; Okada et al., 2009; Chou et al., 2010; Seki et al., 2010). Lateral inhibition, accomplished by inhibitory LNs, has been shown to provide gain control which is defined as a negative feedback loop to keep the AL output in a given range (Olsen et al., 2010). The network of excitatory LNs is providing neuronal excitation between different glomeruli via cholinergic synapses and is assumed to improve odor detection at low intensities (Wilson, 2011).The signal modulation from the input to the output level that we observed in our study for a subset of glomeruli, most likely indicates gain control as well as an increased odor response specificity, allowing for improved odorant component and compound identification and discrimination. Interestingly a previous study by Bhandawat et al. showed that non-linear transformation of olfactory information led to signal broadening in PNs compared to an equivalent number of OSNs using electrophysiological techniques (Bhandawat et al., 2007). Weak OSN input was found to be amplified at the PN level, while strong input was not. Although, seemingly contradictive to our results, our work had the advantage of accounting simultaneously for activity in all OSNs and PNs accessible to our optophysiological technique. Consequently, we were able to investigate and compare more natural proportions of the neuronal populations of OSNs and PNs taking the strong convergence of the sensory input into account. Thus, our study adds complementary information since we investigated the neuronal ~10:1 (10 OSN synapse in average onto 1 PN) relationship between the OSN and PN level. Bhandawat et al.'s study showed stronger PN than OSN responses in 7 glomeruli in response to 18 odors comparing a similar number of OSNs and PNs (1:1). Analyzing the responses of 12 glomeruli (i.e. ~37% of all OSNs labeled by Orco-GAL4) in response to 24 odors of a natural odor source and in natural concentrations, we found that all kinds of inhibitory and excitatory network effects occurred when comparing the OSN with the PN level. It is thus impossible to provide a general rule regarding broadening or sharpening effects during signal transition between OSNs and PNs. Both processes do indeed seem to action in parallel. This observation is well in line with a previous study by Silbering et al. providing evidence for a complex and diverse processing mechanism across different glomeruli in the fly AL (Silbering et al., 2008).

### Mixture interaction occurs only at the output level

In a purely linear model the blend representation would be predicted by a linear sum of the blend components. We used a conservative approach for identifying non-linear blend effects by comparing the response to the banana blend with the response to the strongest individual component for each specific glomerulus (Figure 5) (Deisig et al., 2006). The compound that induces the highest response should also represent the compound exhibiting the highest physiological salience in this glomerulus after blend stimulation. Blend responses lower than the response elicited by the most salient compound would therefore indicate mixture suppression (Silbering and Galizia, 2007). This conservative approach does not allow conclusions regarding synergistic effects which, however, have been shown to be exceedingly rare (Akers and Getz, 1993; Tabor et al., 2004; Silbering and Galizia, 2007). Interestingly, our comparison revealed no significant differences at the OSN level, while we found a significant effect of non-linear interactions in the DM2 glomerulus at the PN level. This result is well in line with the study by Silbering and Galizia (2007) showing that the representation of mixtures in *Drosophila* at the OSN level could rather be predicted from the response pattern of the single components, while mixture responses in PNs revealed strong mixture interactions. This is most likely due to the fact that PN responses are strongly modulated by interglomerular inhibition deriving from a glomerulus-specific network of inhibitory LNs (Wilson and Laurent, 2005; Silbering and Galizia, 2007). In addition, our observed linearity in OSN blend processing has earlier been reported in studies of numerous animal species (Tabor et al., 2004; Deisig et al., 2006; Lin et al., 2006; Carlsson et al., 2007). AL input neurons thus represent a linear blend information assembly, only being tuned by the dose response relationship of individual ORs. Well in line with our study is a recent study by Münch et al. that investigated mixture interactions to binary mixtures of banana compounds in the periphery of the fly olfactory system by performing calcium imaging of Or22a-expressing OSNs on the antenna (Münch et al., 2013) – an OSN population that targets glomerulus DM2 (Couto et al., 2005; Fishilevich and Vosshall, 2005). Münch et al. observed that mixture responses are hypoadditive, i.e. the mixture response was equal to the stronger component confirming our findings.

Interestingly, we could demonstrate that also at the level of the output neurons mixture interactions were surprisingly rare, which might be partially attributed to our conservative analysis. Although other studies have shown that global inhibitory network effects have increasing influence on blend interactions with the number of blend components (Deisig et al., 2006, 2010; Silbering and Galizia, 2007), this might only be true for synthetic mixtures and do not account for naturally occurring blends.

### Similarity shifts between representations of the blend and its single components

The similarity between glomerular activation patterns for all banana components was compared to patterns elicited by the complete banana blend. Assuming that distances between glomerular odor representations correlate with behavioral discrimination (Guerrieri et al., 2005), components with the highest similarity should be perceived as connatural as the banana blend. The Euclidean distances between glomerular activation patterns for component and blend responses at the OSN level showed that isoamyl acetate (a compound typical of banana to the human nose) was ranked highest among all components and suggest it as a key component of the banana blend (Figure 6A, Table 2). Just like the banana blend, isoamyl acetate has been shown to be a highly attractive component for *Drosophila* (Ayyub et al., 1990). Notably, in our analysis the majority of components became more similar to the blend representation at the PN level in comparison to the OSN representations. This result is substantiated by the fact that the average component-blend similarity was significantly higher in PNs compared to OSNs (Figure 6). This similarity change indicates that the functional representation of the individual banana components is modulated at the output level by the AL network. Such a processing mechanism might tune the olfactory system to categorize individual banana components with their naturally occurring odor source and to improve the fly's ability to detect suitable food sources against an environmental odor background. Further experiments are necessary to analyze whether the fly perceives the individual banana components as attractive as the banana blend itself.

### Relevance of glomerulus DM2 for banana perception

Glomerulus DM2 displayed the strongest responses both to single components and to the complete banana blend (Figure 3) which is in accordance with the study by Münch et al. (2013). This glomerulus was in addition the only glomerulus that showed significant mixture suppression at the PN level (Figure 5). We propose that this glomerulus has an important role in eliciting fly attraction to an attractive banana odor blend. Indeed, Semmelhack and Wang showed that innate fly behavior can be mediated by activity in individual glomeruli in the *Drosophila* AL (Semmelhack and Wang, 2009). Moreover, a recent study by Knaden et al. that analyzed the coding of odor valence in the *Drosophila* AL, clearly shows that glomerulus DM2 was significantly stronger activated by attractive odorants (Knaden et al., 2012). Future experiments using flies with an Or22a knock-out will shed further light on the behavioral relevance of this glomerulus regarding attractive natural odor sources.

### The impact of natural odor concentrations using functional GC-I

We combined the proven powers of two well established experimental designs, calcium imaging and GC fragmentation of a natural odor extract. The odor components were used as stimuli and presented to the fly while optophysiological measurements of the different processing levels in the AL were performed. In these functional GC-I experiments we were able to simultaneously investigate the majority of OSNs and PNs, respectively, during a single GC run.

To identify natural blend components which activate OSNs in insects common bioassays like GC-coupled electroantennographic detection (GC-EAD) (Arn and Rauscher, 1975; Struble and Arn, 1984) or GC-coupled single sensillum recordings (GC-SSR) (Stensmyr et al., 2003; Stökl et al., 2010) provided excellent data for odor responses in the periphery. While GC-EAD is a relative simple technique which allows conclusions about component activity in the whole insect antenna, GC-SSR experiments allow in addition the measurement of response profiles of specific sensillum types. The functional GC-I technique emerges as a significant expansion of these classical combined GC/electrophysiology techniques since it offers the investigation of olfactory processing in whole neuronal populations under near-natural conditions, meaning the sensory system can be tested under conditions where behavior is most relevant. It will thus be a highly useful tool in future investigations of insect-insect and insect-plant chemical interactions, and could be extended also to other animal groups.

## Author contributions

Marco Schubert planned and carried out all the experiments; Silke Sachse and Bill S. Hansson together conceived and directed the project; Marco Schubert and Silke Sachse interpreted the results, prepared the figures, and wrote the paper.

### Conflict of interest statement

The authors declare that the research was conducted in the absence of any commercial or financial relationships that could be construed as a potential conflict of interest.

## Supplementary material

Supplementary Movie **Ca^2+^ dynamics of olfactory sensory neurons in the antennal lobe evoked by individual banana components**. *Left*, gray scale delta F movie based on the raw data of a left and right antennal lobe with response patterns of olfactory sensory neurons to different banana compounds. All responses represent changes in glomerular florescence. *Right*, GC profile of a banana extract over 5 min showing the most prominent compounds in the banana headspace extract. As examples the names of four characteristic banana components are given (isoamyl acetate, isobutyl butyrate, isoamyl butyrate and isoamyl valerate). The sliding blue bar in the profile represents the time course of odor stimulation via the GC output while the movie (*left*) shows the synchronized physiological responses of olfactory sensory neurons in the antennal lobes.
